# Beyond QuantiFERON-TB Results, the Added Value of a Weak Mitogen Response

**DOI:** 10.3389/fmed.2022.876864

**Published:** 2022-05-30

**Authors:** Marine Jacquier, Christine Binquet, Catherine Manoha, Sylvain Audia, Anne-Laure Simonet-Lamm, Alice Casenaz, Amadou-Khalilou Sow, Lionel Piroth, Mathieu Blot

**Affiliations:** ^1^Department of Infectious Diseases, Dijon-Bourgogne University Hospital, Dijon, France; ^2^Department of Intensive Care, Dijon-Bourgogne University Hospital, Dijon, France; ^3^Lipness Team, INSERM Research Centre LNC-UMR1231 and LabEx LipSTIC, University of Burgundy, Dijon, France; ^4^CHU Dijon-Bourgogne, INSERM, Université de Bourgogne, CIC 1432, Module Épidémiologie Clinique, Dijon, France; ^5^Department of Virology, Dijon-Bourgogne University Hospital, Dijon, France; ^6^Department of Internal Medicine and Clinical Immunology, Dijon-Bourgogne University Hospital, Dijon, France; ^7^Centre Georges-François Leclerc, Dijon, France

**Keywords:** QuantiFERON, mitogen, infection, outcomes, mortality

## Abstract

**Introduction:**

While QuantiFERON-TB gold (QFT) is frequently used, little attention is paid to the mitogen response. How it could be impacted and associated with outcomes is poorly known.

**Methods:**

Retrospective, case-control study in hospitalized patients who underwent QFT testing in two hospitals between 2016 and 2019. We defined two groups of cases with either negative [interferon (IFN)-γ ≤ 0.5 IU/ml, official threshold] or weak (0.5–2 IU/ml) mitogen response, and one group of controls with normal (>2 IU/ml) mitogen response.

**Results:**

A total of 872 patients were included. An ongoing infection was independently associated with both a negative (RR = 4.34; 95% CI = 2.94–6.41) and a weak mitogen response (RR = 2.44; 95% CI = 1.66–3.58). Among tuberculosis patients, a weak mitogen response was associated with a false-negative QFT result (75%) compared to a normal response (20%). Decreasing mitogen response (normal, weak and negative, respectively) was associated with increasing length of hospital stay [median (interquartile range) 5 (3–13), 11 (5–21) and 15 (10–30) days; *p* < 0.001] and increasing hospital mortality (3, 7, and 15%; *p* < 0.001).

**Conclusion:**

Clinicians should take notice of the mitogen response since IFN-γ concentrations lower than <2 IU/ml were associated with false-negative QFT results in tuberculosis patients, independently associated with ongoing infections, and could be associated with worse prognosis.

## Introduction

One quarter of the world population is potentially affected by a latent tuberculosis infection (LTI) (1). Gamma interferon release assays (IGRAs), which measure *Mycobacterium tuberculosis*-specific CD4 + /CD8 + T cell immunity, are recommended for screening LTI (1). The use of QuantiFERON-TB Gold In-Tube (QFT, Qiagen) testing, which uses IGRA, is increasingly used to screen LTI. The interferon gamma (IFN-γ) response to phytohemagglutinin (PHA) as a mitogen is used as a positive control to confirm the proliferative capacity of patient lymphocytes at the time of sampling (2). A “negative” mitogen response (IFN-γ < 0.5 IU/mL) is considered a “negative” positive control, impeding a conclusive result on QFT (“indeterminate result”). Indeterminate results are obtained in 8 to 29% of patients (3–5). Several factors have been associated with indeterminate QFT results, including immunodeficiency, extreme ages and some chronic diseases (5, 6–10).

While most of the identified factors are related to chronic conditions, little attention has been paid to the factors potentially associated with acute conditions at the time of QFT sampling. For instance, the reported frequency of indeterminate results is higher in septic patients [up to 29% (5)] than in healthy individuals [1.5% (9)]. Such observations suggest that ongoing infection could be a frequent cause of negative mitogen response. However, the association between infection and negative mitogen response among patients tested for a suspicion of LTI or tuberculosis (TB) has not yet been addressed. Infections are the most common cause of lymphopenia in hospitalized patients (10), and functional alterations have been observed in lymphocytes during infections such as sepsis/septic shock (11) and pneumonia (12, 13). This well-known T-cell exhaustion phenotype (including quantitative and qualitative lymphocyte defects) is associated with worse outcomes during sepsis (14). Finally, increasing C-reactive-protein (CRP) levels (15) or neutrophil/lymphocyte ratios are also positively correlated with indeterminate QuantiFERON-TB gold (16). Identifying the situations that are associated with negative QFT performance could avoid unnecessary costs and diagnostic delay. This is of particular interest in the context of TB, for which QFT lacks sensitivity (1). It can be assumed that lymphopenia and T-cell exhaustion, which are frequent in TB (17), explain this lack of sensitivity in connection to an altered mitogen response.

In addition, the clinical relevance of the standard 0.5 IU/mL threshold is a matter of debate, and it has been suggested that a response to PHA with IFN-γ < 10 IU/mL could be considered “possibly altered” rather than “normal” (18).

In order to provide a better understanding of indeterminate QFT, our aim was to assess the factors associated with mitogen response, including ongoing or recent infection. The secondary objectives were to study differences between patients with a weak or a negative mitogen response, the association between mitogen response and clinical outcomes, and the QFT results in TB patients according to mitogen response.

## Materials and Methods

### Study Design and Population

**FIGURE 1:**
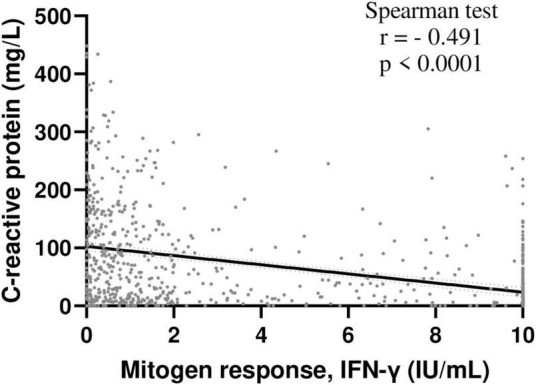
Inverse correlation between INF-γ mitogen response and C-reactive protein levels in hospitalized patients. Correlation between INF-γ mitogen response of QFT and C-reactive protein levels was assessed in hospitalized patients included in the study and with available C-reactive protein data (*n* = 825); Spearman test. NB: IFN-γ, interferon γ, QFT, QuantiFERON-TB Gold.

We conducted a retrospective, observational, case-control study in a cohort of patients hospitalized for at least 48 h in two healthcare facilities [Dijon University hospital and the Georges François Leclerc cancer (CGFL) center] between October 1, 2016 and October 31, 2019, and who underwent QFT testing during hospitalization. Patients under the age of 18, patients only admitted for consultation (as outpatients), those hospitalized for less than 48 h, and those with incomplete QFT results were not included (Figure 1). If a patient underwent several QFTs during the study period, only the first was retained for the analysis.

The patients who met these criteria were classified into three groups according to the mitogen result of the QFT (IFN-γ concentrations). Cases were defined as patients with a “negative” mitogen response, (IFN-γ ≤ 0.5 IU/ml), as defined by the commercial supplier, or with a “weak” mitogen response (IFN-γ = 0.5–2 IU/ml). Controls were defined as patients with a “normal” mitogen response IFN-γ > 2 IU/ml. For each case (either with “negative” or “weak” mitogen response), we included the control patient who had a QFT performed chronologically just after the corresponding case patient (Figure 1). The mitogen threshold of 2 IU/mL was arbitrary defined to obtain balanced groups between negative and weak mitogen response groups.

### QuantiFERON^®^-TB Gold Test

QuantiFERON-TB gold tests were performed by the same laboratory (virology laboratory of the Dijon University Hospital) according to manufacturer instructions. Briefly, four blood collection tubes were drawn: TB1, TB2, and controls (nil and mitogen). One milliliter of whole blood was incubated for 16–24 h at 37°C, after which plasma was harvested and tested using an enzyme-linked immunosorbent assay for the presence of IFN-γ produced in response to the peptide antigens or PHA (mitogen). The nil result was used to adjust for background, heterophile antibody effects or non-specific IFN-γ in blood samples.

### Data Collection

Data were collected from the 3 groups of patients constituted, namely the cases (all the patients with a “negative” or a “weak” mitogen response) and the control patients (1 control patient/case with a “normal” mitogen response).

Clinical and biological data at the time of the QFT test were collected retrospectively. Patients were categorized as immunocompromised if they were treated with an immunosuppressive or corticosteroid therapy (>0.15 mg/kg/day prednisone equivalent during more than 2 weeks or more than to 2 mg/kg per day within 3 months before QFT), or if they had active cancer or hematologic disorder, HIV infection, or primary immune deficiency. Tuberculosis (TB) was recorded if there were associated clinical-radiological signs of TB and it was confirmed with laboratory testing (*Mycobacterium tuberculosis* identification with culture and/or polymerase chain reaction). The presence of an active infection within the last 3 months or at the time of the QFT sampling were recorded if the infection was clinically/biologically suspected or confirmed by the clinician in charge of the patient. Sepsis was considered as severe if requiring ICU admission. Septic shock as sepsis with persisting hypotension requiring vasopressors to maintain mean arterial pressure ≥65 mm Hg and had a serum lactate level >2 mmol/L (18 mg/dL) despite adequate volume resuscitation (19). Primary site and microbial origin of the infection at the time of the QFT were also recorded. Clinical outcomes (onset of an acute infection, sepsis or septic shock after QFT sampling, ICU admission, in-hospital mortality and hospital length of stay) were also recorded.

Study protocol and data collection were registered with the CNIL (*Commission nationale de l’informatique et des libertés*) and are in accordance with French (Loi Informatique et Liberté n°78–17 du 6 janvier 1978) and European (GRPD EU 2016/679) regulations on data protection and patient information (Commitment of compliance MR004 n°2210228 of 3 December 2018) with a waiver of informed consent given the non-interventional study design.

### Statistical Analysis

Continuous variables were expressed as means ± standard deviation (SD) or medians and inter-quartile range (IQR), according to distribution, and categorical variables were expressed as frequencies and percentages. Comparisons were made between the three groups using Kruskal–Wallis tests or ANOVA when appropriate for continuous variables, and the Chi-square test for qualitative variables. When *p*-value was <0.05, *post-hoc* 2 × 2 comparisons were performed using the Student’s *t*-test, Wilcoxon Mann–Whitney test and Chi-square test (or Fisher’s exact test), as appropriate. A False Discovery Rate (FDR) was applied to account for multiple comparisons, and the results are expressed in *Q*-values.

To account for potential confounders, polytomous logistic regressions were fitted considering the normal mitogen response group as the reference. First, a model including only clinical data was fitted, followed by a model including both clinical and biological data. Correlations between variables were checked before their inclusion in the model to avoid colinearity. All variables associated with IFN-γ production with a *p*-value < 0.2 and at least 15 observations in each group were accounted for. Log-linearity was tested for each continuous variable using fractional polynomials (20). A backward step by step selection process was then applied to obtain the final models (one including only clinical variables and a second one including clinical and biological variables). In each final model, interactions between selected variables were systematically tested. Results are expressed as relative risks (RR) and 95% confidence intervals (CI). For variables whose association with mitogen response was non-loglinear, the predicted probabilities of negative or weak response were estimated and plotted against the values of the variables, along with the CIs. A *p*-value < 0.05 was considered statistically significant. Analyses were performed using Stata v15.1 (StataCorp LLC, College Station, TX, United States) and GraphPad Prism software version 9.1.1 (San Diego, CA, United States).

## Results

During the study period, 8,260 QFT were performed, including 2,459 in adult patients hospitalized for at least 48 h (Supplementary Figure 1). Among these patients, 208 (8%) had a “negative” mitogen response (IFN-γ ≤ 0.5 IU/ml) and 228 (9%) had a “weak” mitogen response (IFN-γ = 0.5–2 IU/ml).

Among the 2,023 patients (82%) with a “normal” mitogen response (IFN-γ > 2 IU/ml), 436 were included as controls. Age and sex in the 436 included control patients did not differ significantly from the 1,587 patients who were not selected as controls (59 ± 19 vs. 57 ± 19 years; *p* = 0.06, and 50% female vs. 48%; *p* = 0.54). Finally, 872 patients were included in the study (Supplementary Figure 1).

### Compared Mitogen Responses in the 872 Hospitalized Patients Included

The clinical and biological characteristics of the three groups are compared in Table 1. The “negative” and “weak” mitogen response groups were younger (*p* < 0.001 for both) than “normal” response group and less frequently immunocompromised (*p* < 0.001 for both). The “negative” and “weak” mitogen response groups were not significantly different for these two variables.

**TABLE 1:** Baseline characteristics of patients according to the mitogen response on QFT testing.

	Negative	Weak	Normal	*P*-value	*Q*-value (False Discovery Rate)		
			
	*n* = 208	*n* = 228	*n* = 436		Negative vs. Weak	Negative vs. Normal	Weak vs. Normal
**Demographics**							
Age (years), mean ± SD	62.9 ± 16.7	64.5 ± 18.1	57.3 ± 19.4	<0.001	0.354	<0.001	<0.001
Age (years),				<0.001			
18–45	35 (17)	41 (18)	120 (28)				
46–65	62 (30)	56 (25)	136 (31)				
66–75	60 (29)	58 (25)	96 (22)				
>75	51 (25)	73 (32)	84 (19)				
Male sex, *n* (%)	139 (67)	128 (56)	219 (50)	<0.001	0.044	<0.001	0.148
**Chronic comorbidities**							
Diabetes, *n* (%)	45 (22)	29 (13)	67 (15)	0.033	0.040	0.099	0.357
Chronic heart disease, *n* (%)	69 (33)	53 (23)	87 (20)	0.001	0.042	<0.001	0.323
Chronic kidney disease, *n* (%)	30 (14)	28 (12)	42 (10)	0.184			
Chronic lung disease, *n* (%)	26 (13)	29 (13)	39 (9)	0.217			
Chronic rheumatic disease, *n* (%)	8 (4)	32 (14)	71 (16)	<0.001	<0.001	<0.001	0.447
Chronic liver disease, *n* (%)	14 (7)	14 (6)	15 (3)	0.127			
Auto-immune disease, *n* (%)	62 (30)	83 (36)	170 (39)	0.076			
Immunodeficiency, *n* (%)	121 (58)	118 (52)	158 (36)	<0.001	0.179	<0.001	<0.001
Active tuberculosis, *n* (%)	4 (2)	4 (2)	10 (2)	0.952			
**Events within the last 3 months of the QFT**							
Surgery, *n* (%)	14 (7)	12 (5)	11 (3)	0.031	0.518	0.029	0.133
Infection, *n* (%)	23 (11)	19 (8)	21 (5)	0.013	0.336	0.010	0.141
Severe sepsis, *n* (%)	11 (5)	5 (2)	1 (0.2)	<0.001	0.124	<0.001	0.040
**Clinical status at the time of QFT sampling**							
Temperature > 38.5°C, *n* (%)	92 (44)	72 (31)	79 (18)	<0.001	0,007	<0.001	<0.001
Ongoing infection, *n* (%)	99 (48)	76 (33)	77 (18)	<0.001	0.002	<0.001	<0.001
Severe sepsis, *n* (%)	41 (20)	17 (7)	6 (1)	<0.001	<0.001	<0.001	<0.001
Septic shock, *n* (%)	23 (11)	10 (4)	3 (1)	<0.001	0.009	<0.001	0.002
Bacteremia, *n* (%)	21 (10)	11 (5)	22 (5)	0.028	0.070	0.049	0.901
**Laboratory test results**							
Hemoglobin (g/L), mean ± SD, (*n* = 858)	10.4 ± 1.8	11.3 ± 2.1	12.7 ± 2.0	<0.001	<0.001	<0.001	<0.001
Platelets (x10^9^/l), mean ± SD, (*n* = 859)	302.4 ± 167.0	305.2 ± 144.8	275.6 ± 104.9	0.008	0.853	0.028	0.009
Leukocytes (x10^6^/l), median [Q1; Q3], (*n* = 854)	10,1 [7; 15,8]	9,9 [7,1; 12,6]	7,7 [5,87; 9,4]	<0.001	0,233	0.001	<0.001
Neutrophils (x10^6^/l), median [Q1; Q3], (*n* = 844)	8.57 [5.46; 13.09]	7.04 [4.69; 9.53]	4.53 [3.30; 6.31]	<0.001	0.002	<0.001	<0.001
Neutrophils (x10^6^/l), (*n* = 844)				<0.001			
<1.5	8 (4)	6 (3)	8 (2)				
1.5–4.9	24 (12)	35 (16)	156 (37)				
4.5–9.9	84 (42)	130 (59)	234 (55)				
10–19.9	67 (34)	47 (21)	24 (6)				
≥20	17 (9)	3 (1)	1				
Lymphocytes (x10^6^/l), median [Q1; Q3], (*n* = 844)	0.89 [0.56; 1.44]	1.13 [0.75; 1.54]	1.70 [1.25; 2.33]	<0.001	0.007	<0.001	<0.001
Monocytes (x10^6^/l), median [Q1; Q3], (*n* = 844)	0.74 [0.43; 1.08]	0.69 [0.46; 0.93]	0.64 [0.49; 0.82]	0.041	0.213	0.025	0.092
Eosinophils (x10^6^/l), median [Q1; Q3], (*n* = 844)	0.06 [0; 0.16]	0.12 [0.03; 0.27]	0.15 [0.06; 0.24]	<0.001	<0.001	<0.001	0.048
Creatininemia (μmol/L), median [Q1; Q3], (*n* = 858)	71.0 [53.0; 109.0]	69.0 [54.0; 98.0]	70.0 [57.0; 85.0]	0.942			
C-reactive protein (mg/dL), median [Q1; Q3], (*n* = 749)	96.6 [22.4; 171.0]	53.3 [13.6; 128.0]	11.4 [0.0; 43.7]	<0.001	0.003	<0.001	<0.001
Serum albumin < 30 g/l, *n* (%), (*n* = 825)	171 (88)	139 (71)	111 (31)	<0.001	<0.001	<0.001	<0.001
**QFT results**							
TB antigen–Nil, IFN-γ concentrations ≥ 0.35 IU/mL, *n* (%)	7 (3)	9 (4)	48 (11)	<0.001			
Nil, IFN-γ concentrations < 0.5 IU/mL, *n* (%)	198 (95)	217 (95)	420 (96)	0.703			

*When missing data, the total number of patients with available data is reported. IFN: interferon, SD: standard deviation.*

*Mitogen response: negative (IFN-γ ≤ 0.5 IU/ml), weak (IFN-γ = 0.5–2 IU/ml), and “normal” (IFN-γ > 2 IU/ml).*

As compared to the “normal” mitogen response group, the “negative” response group were significantly more likely to have had surgery and an infection, or a severe infection within the 3 months preceding the QFT, and those with a “weak” response were significantly more likely to have had a severe infection in the 3 months preceding the QFT.

At the time of the QFT sampling, an ongoing infection was detected in, respectively, 18, 33, and 48% of patients with a “normal,” “weak” and a “negative” mitogen response, respectively, and septic shock was detected in 1, 4, and 11%, respectively. Acute infections observed at the time of QFT sampling were mainly bacterial (85%) and of pulmonary origin (56%) (Supplementary Table 1).

The “normal,” “weak” and “negative” mitogen response (respectively) was associated with decreasing hemoglobin, albumin and lymphocyte counts, but increasing neutrophil count and C-reactive protein levels (Table 1). In addition, mitogen response was inversely correlated with C-reactive protein concentrations (Figure 1).

### Factors Independently Associated With “Negative” and “Weak” Mitogen Responses

When considering only clinical variables, multivariable analysis revealed that an ongoing infection at the time of QFT was independently associated with both a “negative” (“negative” vs. “normal” RR = 4.34; 95% CI = 2.94–6.41), and a “weak” mitogen response (“weak” vs. “normal” RR = 2.44; 95% CI = 1.66–3.58), as well as older age and immunodepression. In addition, male sex, diabetes, and an infection within 3 months before QFT were independently associated with a “negative” mitogen response, but not with a “weak” mitogen response, as compared with a “normal” response (Model 1, Table 2). In case of both immunodepression and diabetes, the effect of these both variables was lowered (p for interaction = 0.011 when comparing negative vs. normal mitogen response groups).

**TABLE 2:** Multinomial logistic regression for clinical factors associated with a “negative” and “weak” mitogen response on QFT test (Model 1).

	Negative vs. Normal			Weak vs. Normal		
	RR	95% CI	*p*-value	RR	95% CI	*p*-value
Age (per additional 10 years)	1.12	1.01–1.24	0.027	1.23	1.12–1.35	<0.0001
Sex (male)	1.62	1.12–2.35	0.010	1.14	0.81–1.59	0.460
Diabetes (Yes vs. No)	2.21	1.21–4.06	0.010	0.81	0.43–1.52	0.509
Immunodepression (Yes vs. No)	3.54	2.35–5.33	<0.0001	2.08	1.44–3.00	<0.0001
Ongoing infection at the time of QFT (Yes vs. No)	4.34	2.94–6.41	<0.0001	2.44	1.66–3.58	<0.0001
Infection within 3 months before QFT (Yes vs. No)	2.15	1.10–4.20	0.025	1.75	0.89–3.42	0.103
Interaction diabetes × immunodepression	0.30	0.12–0.75	0.011	0.55	0.21–1.46	0.227

*No missing data for the reported variables (872 patients are considered in the analysis). Mitogen response: negative (IFN-γ ≤ 0.5 IU/ml), weak (IFN-γ = 0.5–2 IU/ml), and “normal” (IFN-γ > 2 IU/ml). RR, relative risk; CI, confidence interval.*

When the “severe infection at the time of the QFT” variable was added in the model instead of the “an ongoing infection at the time of QFT” variable (model 2), the first variable was independently associated with both a “negative” (RR = 20.09; 95% CI = 8.69–46.46) and a “weak” (RR = 7.10; 95% CI = 2.93–17.23) mitogen response (Supplementary Table 2).

When considering both clinical and biological variables (model 3), multivariable analysis revealed that lower serum albumin, hemoglobin and lymphocytes, but higher neutrophil counts were independently associated with both a “negative” and “weak” mitogen responses (vs. “normal”). In addition, sex, immunodepression and higher platelets were independently associated with a “negative” mitogen response, and diabetes was associated with a “weak” mitogen response when compared to the “normal” mitogen response group (Supplementary Table 3 and Supplementary Figure 2).

When the variable “a severe infection at the time of the QFT” was added in the model instead of “an ongoing infection at the time of QFT” (model 4), this first was independently associated with a “negative” mitogen response (RR = 2.78; 95% CI [1.03–7.51]) (Supplementary Table 4 and Supplementary Figure 3).

### QuantiFERON-TB Gold Results in Patients With Confirmed Tuberculosis

Among the 872 patients included, 18 patients (2%) had microbiologically confirmed TB, among whom 6 (33%) had a false negative QFT [2/10 (20%) with a “normal” mitogen response, 3/4 (75%) with a “weak” and 1/4 (25%) with a “negative” mitogen response (Supplementary Table 6)]. Interestingly, one patient underwent serial QFT. The first test at the time of TB diagnosis (pulmonary and peritoneal involvement) was considered negative (Ag TB-Nil [IFN-γ] = 0.05 IU/mL), while the mitogen response was “weak” (mitogen [IFN-γ] = 1.03 IU/mL). The second one, 5 weeks after the start of antimycobacterial treatment, was positive (Ag TB-Nil [IFN-γ] = 3.86 IU/mL). This positive result was associated with an improved mitogen response (mitogen [IFN-γ] > 10 IU/mL), decreased C-reactive protein levels, and no change in the lymphocyte count (Supplementary Figure 4).

### Clinical Outcomes According to the Mitogen Response

**FIGURE 2:**
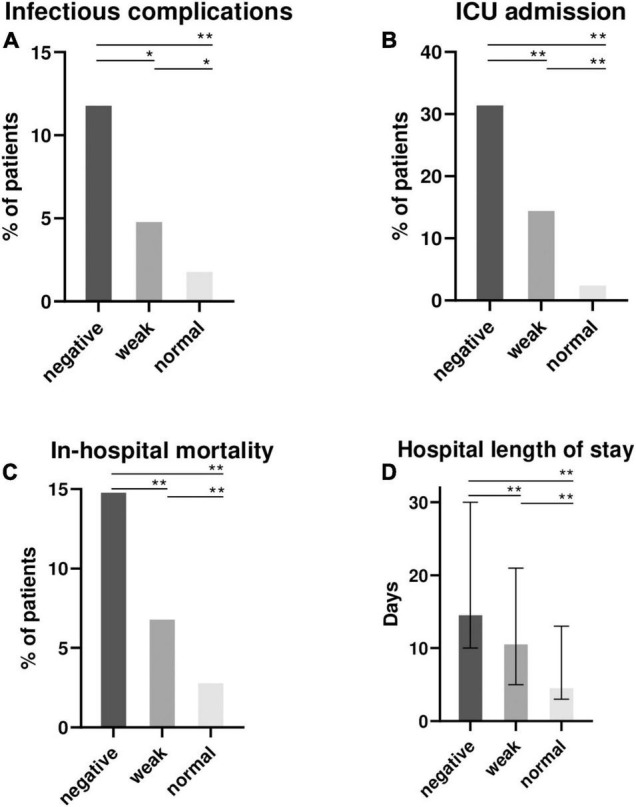
Clinical outcomes of 872 hospitalized patients according to the mitogen response of QFT (negative, weak or normal). Proportion of patients with infectious complications during hospital stay after QFT sampling **(A)**, intensive care unit (ICU) admissions **(B)**, in-hospital mortality **(C)** and the median (interquartile range) hospital length of stay **(D)** were recorded and represented according to the mitogen response: negative (IFN-γ ≤ 0.5 IU/ml), weak (IFN-γ = 0.5–2 IU/ml) and “normal” (IFN-γ > 2 IU/ml). NB: QFT, QuantiFERON-TB Gold; ICU, intensive care unit; IFN-γ, interferon γ. Comparisons were made between the 3 groups using Kruskall-Wallis test for continuous variable, and the Chi-square test for qualitative variables. *Post-hoc* 2 × 2 comparisons were performed using the Wilcoxon Mann-Whitney test and Chi-square test as appropriate. False Discovery Rate *post hoc* correction for multiple comparisons was used and *P* value reported in the figure : *P < 0.05; **P < 0.01.

The mitogen response (respectively “normal,” “weak” and “negative”) was associated with an increasing proportion of patient with onset of infectious complications (2, 5, 12%; *p* < 0.001), ICU admissions (3, 15, 32%; *p* < 0.001), hospital length of stay [median (interquartile range) = 5 (3–13); 11 (5–21) and 15 (10–30) days; *p* < 0.001] and in-hospital mortality (3%, 7% and 15%; *p* < 0.001) (Figure 2 and Supplementary Table 6).

## Discussion

Our case-control study involving 872 hospitalized patients who underwent QFT testing yielded four main results. First, the presence of an ongoing infection at the time of QFT was independently associated with a “negative” mitogen response and therefore indeterminate QFT results. Second, patients with a “weak” mitogen response and “negative” mitogen response had several similarities. Third, among confirmed TB patients, a “weak” mitogen response was associated with false negative QFT results. Fourth, the mitogen response was associated with hospital length of stay and in-hospital mortality.

In our study, 8% of hospitalized patients had indeterminate QFT results, which is in the range of other previously published studies (5). Multivariable analyses identified age, immunodepression, serum albumin, hemoglobin, and neutrophil and lymphocyte counts as factors associated with a negative mitogen response, as previously reported (3, 7). However, interestingly, we found that both recent and ongoing infection were independent factors associated with a “negative” mitogen response. Infections are frequent in hospitalized patients, but our results suggest QFT should not be performed until after recovery. Infections, and particularly severe sepsis, are associated with both lymphopenia and functional lymphocyte defects (T-cell exhaustion or anergy), which resolve at least partially about 1 month after sepsis (21, 22).

We then addressed the problem of the cut-offs for mitogen positivity. We observed that patients with “negative” and “weak” mitogen responses have similar characteristics, especially for immunodepression status, which was identified in, respectively, 58 and 52% of these patients, compared with 36% of patients with “normal” response. Our results raise the question of whether a LTI can be confidently interpreted with a “weak” mitogen response. Seeing that such a result is not a “normal” response to PHA stimulation, it may be thought to be a false negative QFT when diagnosing LTI, or even TB. Furthermore, our data for patients with microbiologically confirmed TB are illustrative. QFT was less frequently positive if mitogen response was “weak” (25% of cases) compared to “normal” (80% of cases). In addition, in the patient who underwent serial QFT, there was a correction of both mitogen and TB antigen responses between TB diagnosis and 5 weeks later under treatment.

These results offer new insight about QFT performance, particularly during TB, which is associated with both systemic inflammation and T-cell exhaustion. One could assume that decrease in both lymphocyte activation and systemic inflammation (reflected by CRP levels) would improve lymphocyte function. The exhaustion of T lymphocytes is a well-known consequence of sepsis (23) and other chronic infections such as TB (17, 24), minimizing the damage to host tissue related to inflammatory response (25). This manifests through attenuated cell proliferation, impaired cytotoxic function, and attenuated IL-2 and IFN-γ production (23). The negative correlation we observed between C-reactive protein and mitogen response further supports this assumption. In the context of culture-confirmed TB, QFT testing has been associated with negative sensitivity (∼70%) (26, 27). If performed in such a context, clinicians must interpret QFT with caution, especially if mitogen response if weak (IFN-γ = 0.5–2 IU/ml), whatever the “official” interpretation. In addition, the contribution of the two tubes TB1 and TB2, evaluating, respectively, the T-CD4 and T-CD8 responses, did not allow to mitigate this limitation since both T-CD4 and CD8 are non-selectively affected by T-cell exhaustion during sepsis (23).

Finally, in hospitalized patients who underwent a QFT, we observed that weaker IFN-γ production was associated with a worse prognosis, considering infectious complications, ICU admission, in-hospital mortality, and hospital length of stay. Huang et al. previously reported that a lower mitogen response was associated with worse 1-year survival in TB patients (27). However, our study is novel in that it is the first study to observe an association between mitogen response and clinical outcomes in hospitalized patients. We can assume that confirmed TB cases with extensive diseases and/or those with systemic involvement were associated with both systemic inflammation and T-cell exhaustion. If QFT is performed, the mitogen response may serve as a surrogate biomarker for assessment of immune competence in hospitalized patients whose determinants are varied (old age, immunodepression, previous or current infection, which were associated with “negative” or “weak” mitogen response in our study).

Our study has several limitations. First, the retrospective design led to missing data, mainly for laboratory results. In addition, this was a single center study when considering the laboratory which performed QFT, and the results may not be reproducible in other settings. However, the laboratory is accredited for QFT analysis (COFRAC accreditation ISO15189 n°1–8125). Third, the cutoff we used to define “weak” mitogen response is rather arbitrary, and whether higher thresholds would be associated with different parameters and outcomes was not assessed.

In conclusion, it appears to be of utmost interest to consider the mitogen response in hospitalized patients who undergo QFT, in particular when this response is “negative” or “weak” (IFN-γ ≤ 2 IU/mL). In suspected TB, aside from the response to the TB antigen, it suggests the need for further testing to avoid premature interpretation and misdiagnosis. Outside the scope of TB, the “weak” mitogen response was independently associated with ongoing infections and could be associated with a worse prognosis. The IFN-γ response to mitogen and the threshold of 2 IU/mL may thus serve as a prognostic biomarker in hospitalized patients, even though further confirmation in other cohorts is needed.

## Data Availability Statement

The original contributions presented in the study are included in the article/Supplementary Material, further inquiries can be directed to the corresponding author.

## Ethics Statement

Ethical review and approval was not required for the study on human participants in accordance with the local legislation and institutional requirements. Written informed consent for participation was not required for this study in accordance with the national legislation and the institutional requirements.

## Author Contributions

MJ, MB, CB, and LP: concept and design. MJ, MB, and A-LS-L: recruitment of patients. MJ, MB, A-KS, CB, and LP: acquisition, analysis, or interpretation of data. MJ, MB, and CB: drafting of the manuscript. MJ, CB, CM, SA, A-LS-L, AC, A-KS, LP, and MB: critical revision. CB and MB: supervision. All authors contributed to the article and approved the submitted version.

## Conflict of Interest

The authors declare that the research was conducted in the absence of any commercial or financial relationships that could be construed as a potential conflict of interest.

## Publisher’s Note

All claims expressed in this article are solely those of the authors and do not necessarily represent those of their affiliated organizations, or those of the publisher, the editors and the reviewers. Any product that may be evaluated in this article, or claim that may be made by its manufacturer, is not guaranteed or endorsed by the publisher.

## Abbreviations

CRP: C-reactive -protein
FDR: false discovery rate
HIV: human immunodeficiency virus
ICU: intensive care unit
IGRAs: interferon gamma release assays
IFN- γ: interferon gamma
LTI: latent tuberculosis infection
PHA: phytohemagglutinin
QFT: QuantiFERON-TB gold
TB: tuberculosis.
