# Supplementary material for: Beyond QuantiFERON-TB Results, the Added Value of a Weak Mitogen Response
Source: Front Med (Lausanne). 2022 May 30;9:876864. doi: 10.3389/fmed.2022.876864 (PMC9197320; doi:10.3389/fmed.2022.876864)
Supplement: Supplementary file 1 [file Data_Sheet_1.pdf]

Beyond QuantiFERON-TB results, the added value of a weak mitogen response.

-

Supplemental data

Supplemental table 1: Characteristics of infections diagnosed at the time of QFT sampling according to mitogen response.

	**negative n = 208**	**weak n = 228**	**normal n = 436**	***p*-value**
**Infection type**				**< 0.001**
Bacteria, n (%)	83 (40)	63 (28)	65 (15)	
Virus, n (%)	8 (4)	6 (3)	5 (1)	
Fungus, n (%)	7 (3)	6 (3)	2	
Parasite, n (%)	0	1	3 (1)	
**Primary infection site**				**< 0.001**
Lung, n (%)	55 (26)	41 (18)	22 (5)	
Urinary tract, n (%)	7 (3)	3 (1)	5 (1)	
Digestive, n (%)	12 (6)	7 (3)	10 (2)	
Other sites, n (%)	25 (12)	22 (10)	39 (9)	

Notes: Mitogen response: negative (IFN-γ ≤ 0.5 IU/ml), weak (IFN-γ = 0.5-2 IU/ml) and "normal" (IFN-γ > 2 IU/ml).

Supplemental table 2: Multinomial logistic regression for clinical factors associated with poor and low mitogen response in QFT test (Model 2)

	negative vs. normal			weak vs. normal		
	**RR**	**95% CI**	***p*-value**	**RR**	**95% CI**	***p*-value**
**Age (per additional 10 years)**	1.15	1.04 - 1.27	0.008	1.24	1.13 - 1.36	< 0.0001
**Sex (male)**	1.62	1.12 - 2.34	0.011	1.17	0.84 - 1.64	0.349
**Diabetes (Yes vs. No)**	2.12	1.14 - 3.93	0.017	0.79	0.42 - 1.49	0.463
**Immunodepression (Yes vs. No)**	3.41	2.26 - 5.15	<0.0001	1.98	1.38 - 2.84	< 0.0001
**Severe infection (Yes vs. No)**	20.09	8.69 - 46.46	<0.0001	7.10	2.93 - 17.23	< 0.0001
**Interaction diabetes x immunodepression**	0.32	0.13 - 0.82	0.018	0.60	0.23 - 1.58	0.302

Notes: No missing data for the reported variables (872 patients are considered in the analysis).

Mitogen response: negative (IFN-γ ≤ 0.5 IU/ml), weak (IFN-γ = 0.5-2 IU/ml) and “normal” (IFN-γ > 2 IU/ml).

Abbreviation: RR= relative risk, CI: confidence interval

Supplemental table 3: Multinomial logistic regression considering clinical and biological factors associated with a poor and low mitogen response in QFT test (Model 3)

	negative vs. normal			weak vs. normal		
	**RR**	**95% CI**	***p*-value**	**RR**	**95% CI**	***p*-value**
**Sex (male)**	2.71	1.63 - 4.52	<0.0001	1.42	0.92 - 2.19	0.109
**Diabetes (Yes vs. No)**	1.22	0.68 - 2.19	0.504	0.53	0.29 - 0.95	0.034
**Immunodepression (Yes vs. No)**	2.17	1.32 - 3.56	0.002	1.45	0.95 - 2.22	0.086
**Serum albumin < 30g/L (Yes vs. No)**	3.29	1.78 - 6.11	<0.0001	1.98	1.22 - 3.23	0.006
**Hemoglobin (g/dL)**	0.71	0.62 - 0.81	<0.0001	0.83	0.74 - 0.94	0.002
**Platelets (x10^3/mm3)**	1.003	1.001 - 1.005	0.003	1.00	0.99 - 1.003	0.059
**Neutrophils (/mm3)**	Cf suppl. figure 2A		<0.0001	Cf suppl. figure 2B		<0.0001
**Lymphocytes (/mm3)**	Cf suppl. figure 2C		<0.0001	Cf suppl. figure 2D		<0.0001

Notes: Due to missing data in the reported variables, 692 patients are considered in the analysis.

Mitogen response: negative (IFN-γ ≤ 0.5 IU/ml), weak (IFN-γ = 0.5-2 IU/ml) and “normal” (IFN-γ > 2 IU/ml).

Abbreviation: RR= relative risk, CI: confidence interval

Supplemental table 4: Multinomial logistic regression with clinical and biological factors associated with a poor and low mitogen response in QFT test (Model 4)

	negative vs. normal			weak vs. normal		
	**RR**	**95% CI**	***p*-value**	**RR**	**95% CI**	***p*-value**
**Sex (yes = man)**	2.52	1.50 - 4.22	<0.0001	1.39	0.90 - 2.16	0.135
**Diabetes (Yes vs. No)**	1.23	0.68 - 2.21	0.498	0.52	0.29 - 0.95	0.032
**Immunodepression (Yes vs. No)**	2.28	1.38 - 3.77	0.001	1.44	0.94 - 2.21	0.091
**Albuminemia < 30g/L (Yes vs. No)**	3.13	1.68 - 5.82	<0.0001	1.97	1.21 - 3.21	0.007
**Hemoglobin (g/dL)**	0.72	0.63 - 0.83	<0.0001	0.84	0.74 - 0.95	0.004
**Platelets (x10^3/mm3)**	1.003	1.001 - 1.005	0.002	1.00	0.999 - 1.003	0.07
**Neutrophils (/mm3)**	Cf suppl. figure 3A		<0.0001	Cf suppl. figure 3B		<0.0001
**Lymphocytes (/mm3)**	Cf suppl. figure 3C		<0.0001	Cf suppl. figure 3D		<0.0001
**Severe infection**	2.78	1.03 - 7.51	0.043	1.58	0.56 - 4.47	0.387

Notes: Due to missing data, 692 patients are considered in the analysis

Mitogen response: negative (IFN-γ ≤ 0.5 IU/ml), weak (IFN-γ = 0.5-2 IU/ml) and “normal” (IFN-γ > 2 IU/ml).

Abbreviation: RR= relative risk, CI: confidence interval

Supplemental table 5. QFT results in patients with proven tuberculosis.

	**negative n=4**	**weak n=4**	**normal n=10**
False negative QFT test, n (%)	1 (25)	3 (75)	2 (20)
IFN-γ concentrations (*M.tuberculosis* antigens stimulation), mean ± SD	10.5 ± 14.6	0.2 ± 0.3	6.9 ± 8.5

Notes: Mitogen response: negative (IFN-γ ≤ 0.5 IU/ml), weak (IFN-γ = 0.5-2 IU/ml) and "normal" (IFN-γ > 2 IU/ml).

Abbreviations: SD: standard deviation, IFN: interferon, IQR: interquartile range, QFT: QuantiFERON Tuberculosis

Supplemental table 6: Clinical outcomes in hospitalized patients according to mitogen response in QFT test.

	negative	weak	normal		Q-value (False Discovery Rate)		
	n = 208	n = 228	n = 436	*p*-value	negative vs. weak	negative vs. normal	weak vs. normal
Infectious complication, n (%)	25 (12)	12 (5)	9 (2)	< 0.001	0.017	0.003	0.025
Severe sepsis, n (%)	11 (5)	5 (2)	1	< 0.001	0.086	0.003	0.017
Intensive care unit admission, n (%)	67 (32)	35 (15)	13 (3)	< 0.001	0.001	0.001	0.001
Hospital length of stay (days), median (IQR)	15 (10-30)	11 (5-21)	5 (3-13)	< 0.001	0.001	0.001	0.001
In-hospital mortality, n (%)	32 (15)	17 (7)	13 (3)	< 0.001	0.009	0.003	0.009

Notes: Mitogen response: negative (IFN-γ ≤ 0.5 IU/ml), weak (IFN-γ = 0.5-2 IU/ml) and “normal” (IFN-γ > 2 IU/ml).

Abbreviations: IQR: interquartile range, QFT : QuantiFERON Tuberculosis

Supplemental Figure 1.

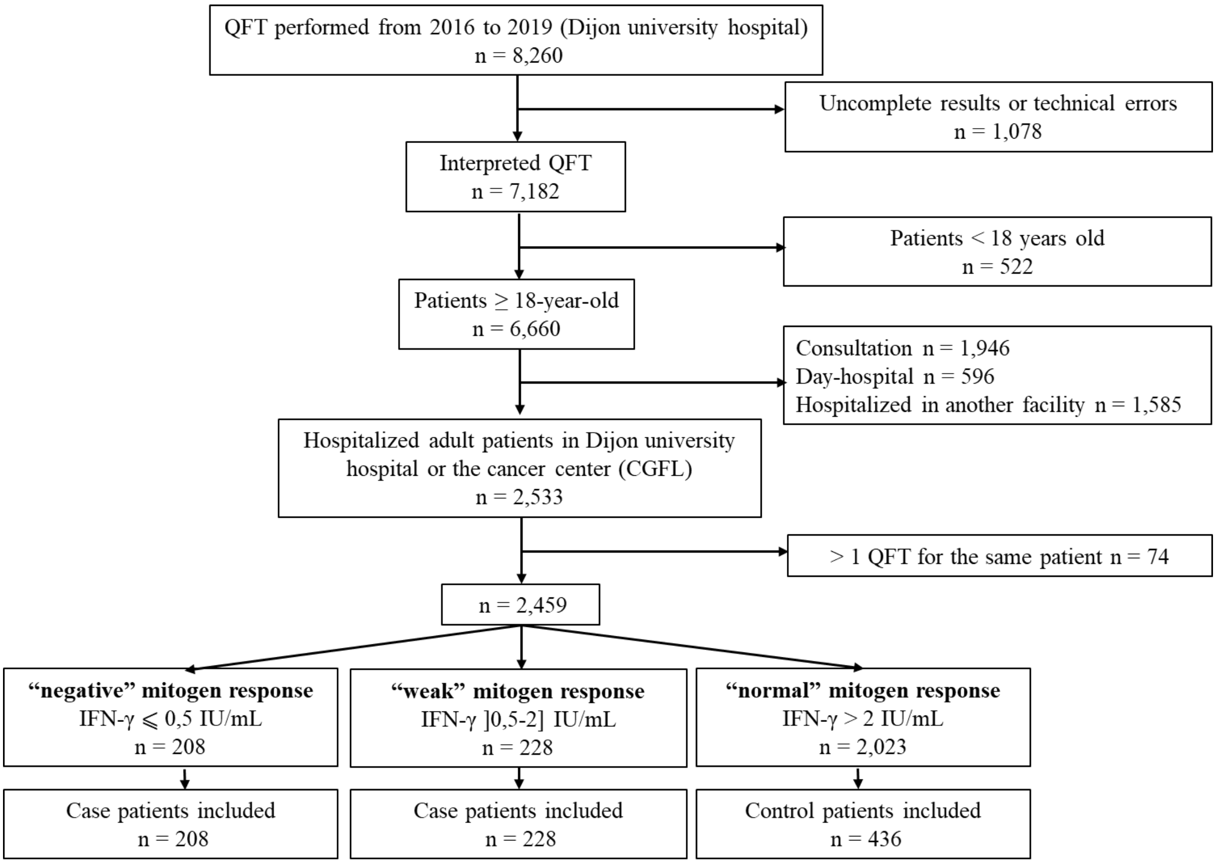

Supplemental Figure 1. Flowchart

Abreviations: IFN-γ = interferon γ, QFT = QuantiFERON-TB Gold

Supplemental Figure 2.

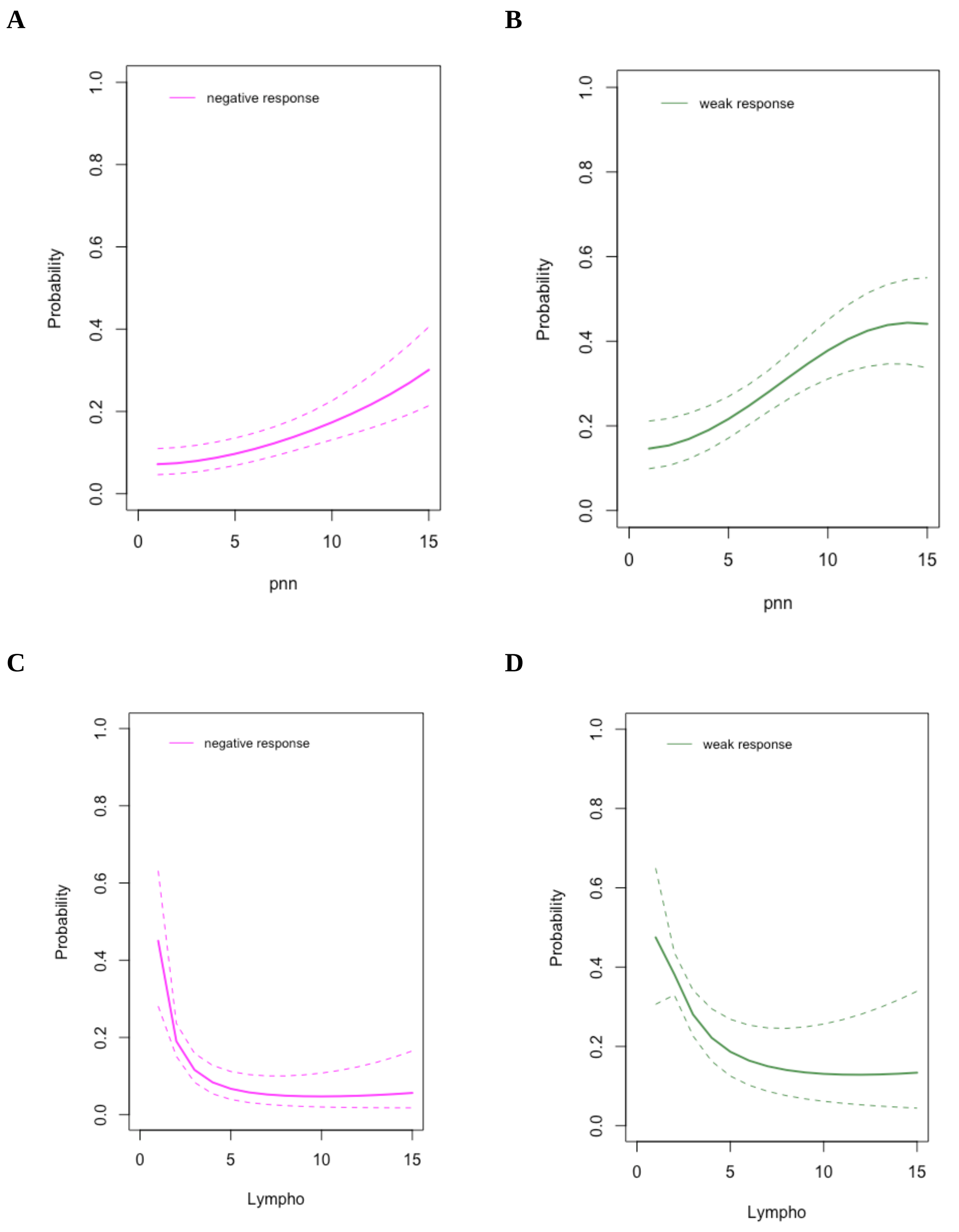

Supplemental Figure 2: Predicted probability of having a poor (A) and low (B) mitogen response according to neutrophil count, and predicted probability of having a poor (C) and low (D) mitogen response according to the lymphocyte count (multinomial logistic regression, Model 3, Supplemental table 3).

Supplemental Figure 3.

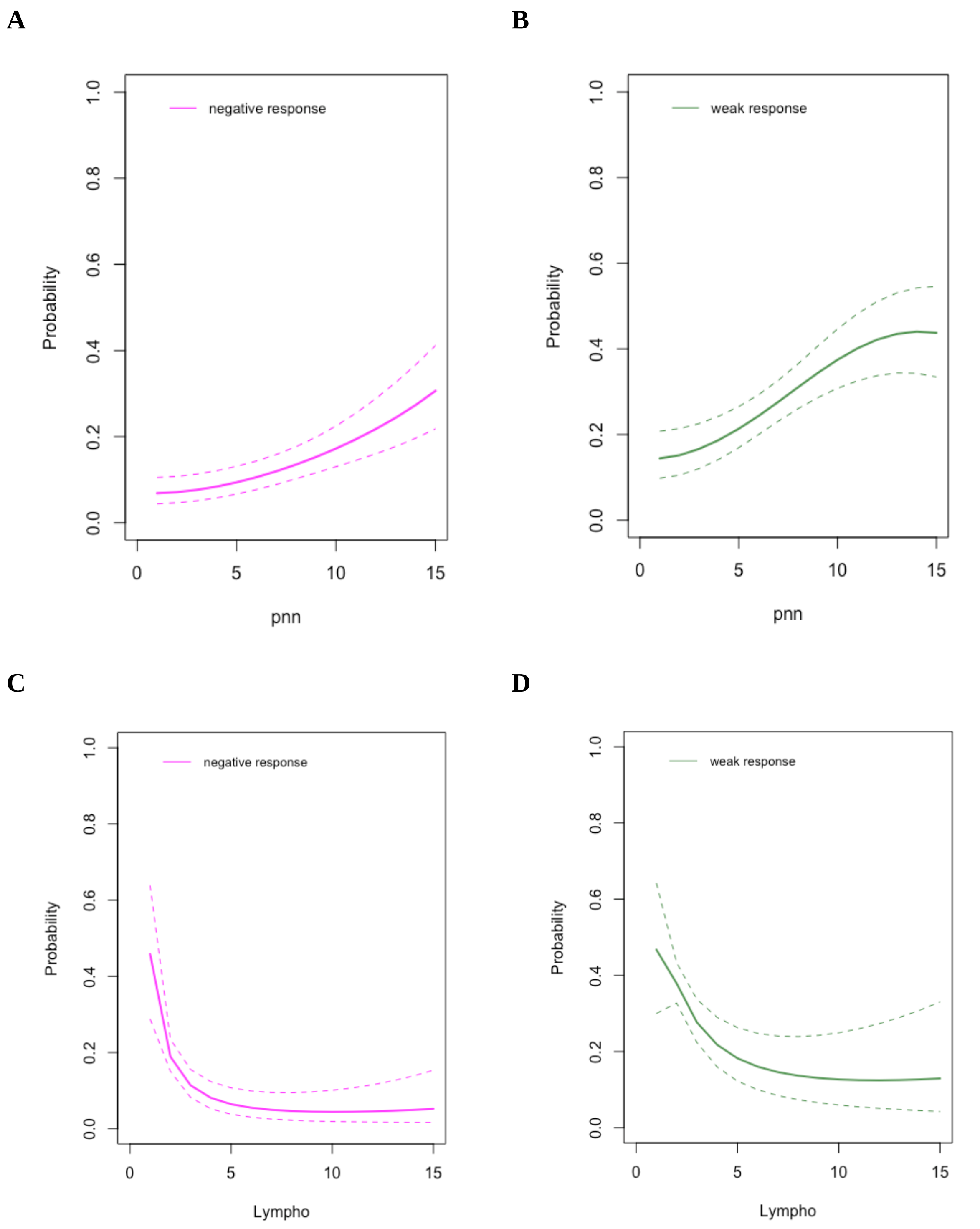

Supplemental Figure 3: predicted probability of having a poor (A) and low (B) mitogen response according to neutrophils count, and predicted probability of having a poor (C) and low (D) mitogen response according to lymphocyte count (multinomial logistic regression, Model 4, Supplemental table 4).

Supplemental Figure 4.

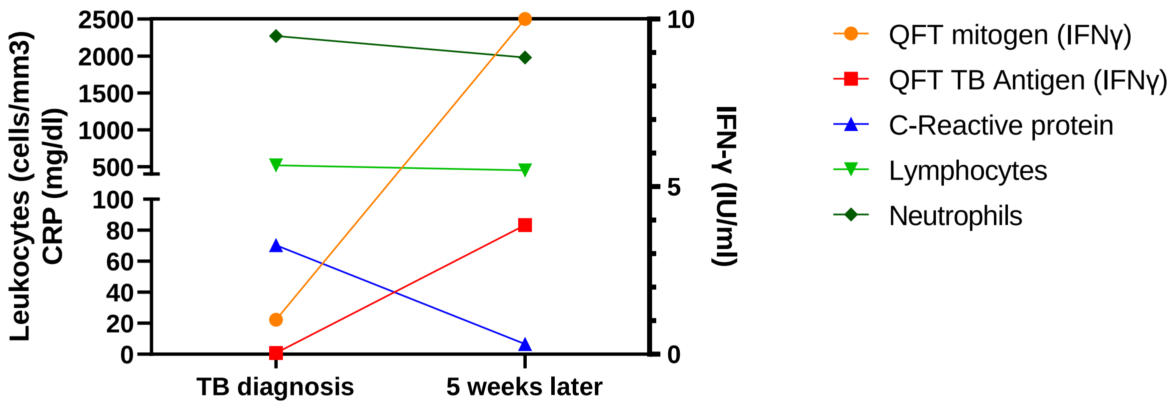

Supplemental Figure 4: Case of a patient who underwent serial QFT testing in a context of microbiologically proven TB.
